# Molybdenum anode: a novel electrode for enhanced power generation in microbial fuel cells, identified via extensive screening of metal electrodes

**DOI:** 10.1186/s13068-018-1046-7

**Published:** 2018-02-13

**Authors:** Takahiro Yamashita, Hiroshi Yokoyama

**Affiliations:** Division of Animal Environment and Waste Management Research, Institute of Livestock and Grassland Science, National Agriculture and Food Research Organization (NARO), 2 Ikenodai, Tsukuba, 305-0901 Japan

**Keywords:** *Geobacter*, Metal anode, Metal cycle, Microbial fuel cell, Molybdenum trioxide, Tungsten trioxide

## Abstract

**Background:**

Metals are considered a suitable anode material for microbial fuel cells (MFCs) because of their high electrical conductivity. However, only a few types of metals have been used as anodes, and an extensive screening of metals has not yet been conducted. In this study, to develop a new metal anode for increased electricity generation in MFCs, 14 different metals (Al, Ti, Fe, Ni, Cu, Zn, Zr, Nb, Mo, Ag, In, Sn, Ta, and W) and 31 of their oxidized forms were comprehensively tested. Oxidized-metal anodes were prepared using flame oxidation, heat treatment, and electrochemical oxidation. The selected anodes were further evaluated in detail using air–cathode single-chambered MFCs.

**Results:**

The untreated Mo and electrochemically oxidized Mo anodes showed high averages of maximum power densities in the screening test, followed by flame-oxidized (FO) W, FO-Fe, FO-Mo, and Sn-based anodes. The untreated Mo and FO-W anodes were selected for further evaluation. X-ray analyses revealed that the surface of the Mo anode was naturally oxidized in the presence of air, forming a layer of MoO_3_, a known oxidation catalyst. A high maximum power density (1296 mW/m^2^) was achieved using the Mo anode in the MFCs, which was superior to that obtained using the FO-W anode (1036 mW/m^2^). The Mo anode, but not the FO-W anode, continued to produce current without detectable corrosion until the end of operation (350 days). *Geobacter* was abundant in both biofilms on the Mo and FO-W anodes, as analyzed by high-throughput sequencing of the *16S rRNA* gene.

**Conclusions:**

The screening test revealed that Mo, W, Fe, and Sn are useful MFC anode materials. The detailed analyses demonstrated that the Mo anode is a high-performance electrode with structural simplicity and long-term stability in MFCs. The anode can be easily prepared by merely shaping Mo materials to the desired forms. These properties would enable the large-scale preparation of the anode, required for practical MFC applications. This study also implies the potential involvement of *Geobacter* in the Mo and W cycles on Earth.

**Electronic supplementary material:**

The online version of this article (10.1186/s13068-018-1046-7) contains supplementary material, which is available to authorized users.

## Background

Microbial fuel cells (MFCs) are promising bioelectrochemical reactors that produce electricity directly from organic wastewater, with microbes as the catalyst [1–3]. Since MFCs can be used to simultaneously purify the wastewater, the application of MFCs in an energy self-sufficient domestic wastewater treatment has attracted [4]. In MFCs, exoelectrogenic bacteria adhere to the anode surface, and decompose the organic matter in wastewater to electrons, H^+^, and CO_2_ under anaerobic conditions. The bacteria transfer the electrons to the anode and the electrons flow to the cathode via an external circuit. The electron transfer to the anode is a key reaction that defines the theoretical limits of MFC energy conversion [5]. The anode material can act as a catalyst for the transfer reaction while maintaining the conductivity [6]. Therefore, the development of new anode materials to facilitate the reaction is imperative for improving the power output of MFCs.

Carbon-based anodes, such as carbon paper, carbon cloth, and carbon brush, have been commonly used in MFCs because of their chemical stability, large effective surface areas, and high biocompatibility with microbes [7]; on the other hand, their electrical conductivity is typically two to three orders of magnitude lower than that of metal electrodes [8]. Sophisticated carbonaceous anodes with nanoscale structures have been developed over the past decade; e.g., a carbon nanotube-coated anode [9] and polyaniline-modified 3D graphene anode [10] promote electricity generation in MFCs owing to increased surface area and enhanced biofilm formation. While metal-based anodes are not commonly used in MFCs, some fine-structure metal anodes have been developed, e.g., a stainless-steel (SS) anode decorated with graphenes [11] and a Ni foam anode coated with polyaniline/TiO_2_ [12]. Such elaborate anodes are characterized by high current-generating performances. However, the preparation process of these anodes is substantially complicated, and expensive materials, such as carbon nanotubes, are frequently required. Therefore, these anodes would not be suitable for large-scale MFC applications, such as wastewater treatment, including sewage processing. Easily prepared, highly conductive, and cost-effective anodes are needed for such large-scale applications.

Unmodified metals are rarely used as anode material in MFCs, because their biocompatibility with microbes is considered to be low, resulting in a diminished power output. Recently, it was reported that exoelectrogenic bacteria can form biofilms on the surfaces of SS and Cu anodes [8, 13, 14]. A new method for flame-oxidizing the SS anode surface has been reported to improve the current output in bioelectrochemical systems [15] and power generation in MFCs [16]. The power output using the flame-oxidized (FO) SS anode was 24% higher than that of a common carbon-cloth anode in MFCs. Furthermore, *Geobacter* spp., well-characterized exoelectrogenic bacteria, were more abundant in a biofilm community formed on the FO-SS anode than in one formed on the carbon-cloth anode [16]. *Geobacter* spp. adhere to Fe(III)-oxide particles and can transfer electrons to Fe(III) via *c*-type cytochromes on their cell walls [17]. Flame oxidation of the SS anode results in the formation of Fe(III)-oxide particles on the anode surface; it has been suggested that these particles concentrate *Geobacter* spp. on the anode surface [16].

Until now, Fe has been mainly used as the material of metal-based anodes in MFCs, because most exoelectrogenic bacteria possess Fe(III) oxide-reducing activity. However, in addition to Fe(III), other metals, including Cr(VI), Mn(IV), Cu(II), Mo(VI), Ag(I), Au(III), and U(VI), are also reduced by bacteria [18, 19]. This suggests that not only Fe-based anodes but also other metal-based anodes might achieve a high current-generating performance in MFCs; nevertheless, extensive screening of metals and oxidized metals as the anode material has not been conducted. In the current study, 45 types of metal-based anodes were comprehensively tested, and the selected anodes were further evaluated in detail for their possible application in MFCs.

## Methods

### Metal anode preparation

Metal plates and meshes used in this study were purchased from Nilaco Co. (Tokyo, Japan) and Clever Co. (Aichi, Japan), respectively. To prepare FO anodes, the metal anodes were flamed (> 1200 °C) with a kitchen stovetop burner using natural gas as the fuel [16]. The treatment time for each metal anode is shown in Table 1. Heat treatment (HT) of the metal anodes was conducted in the presence of air using a muffle furnace. The metal was placed in the preheated furnace for the indicated time period (Table 1); it was subsequently removed to cool down to ambient temperature. Electrochemically oxidized (EO) anodes were prepared in acidic (EO_acid_) or alkaline (EO_alk_) electrolysis solutions. The metal anode and a Pt counter electrode were connected to the positive and negative terminals, respectively, of a direct current power supply. The treatment time, composition of the electrolysis solutions, and applied voltage for each metal are shown in Table 1.

**Table 1:** Conditions for the preparation of oxidized-metal anodes

Anode	Oxidation conditions
EO_acid_-Al	10% H_2_SO_4_ at 5 V for 5 min
HT-Al	620 °C for 6 h
EO_acid_-Ti	0.1 M H_2_SO_4_ at 20 V for 1 min
FO-Ti	2 min
EO_alk_-Fe	10 M NaOH at 5 V for 10 min
FO-Fe	10 min
EO_acid_-Ni	10% H_2_SO_4_ at 2.5 V for 1 min
EO_alk_-Ni	10 M NaOH at 5 V for 30 min
FO-Ni	2 min
EO_acid_-Cu	20% H_2_SO_4_ at 2.5 V for 1 min
FO-Cu	2 min
EO_alk_-Zn	4 M NaOH at 2.5 V for 5 min
HT-Zn	800 °C for 5 min
EO_alk_-Zr	1% NaOH at 10 V for 1 min
FO-Zr	2 min
EO_acid_-Nb	0.1 M H_2_SO_4_ at 20 V for 1 min
FO-Nb	2 min
EO_acid_-Mo	0.2 M acetic acid at 30 V for 20 min
EO_alk_-Mo	1% NaOH at 3 V for 10 min
FO-Mo	10 min
EO_acid_-Ag	1 M HCl at 3 V for 1 min
EO_alk_-Ag	1% NaOH at 5 V for 1 min
HT-Ag	920 °C for 11 h
EO_acid_-In	0.1 M H_2_SO_4_ at 5 V for 1 min
EO_acid_-Sn	0.1 M H_2_SO_4_ at 5 V for 1 min
EO_alk_-Sn	1% NaOH at 10 V for 1 min
HT-Sn	190 °C for 3 h
EO_acid_-Ta	0.1 M H_3_PO_4_ at 10 V for 10 min
EO_acid_-W	0.1 M H_2_SO_4_ at 8 V for 5 min
EO_alk_-W	1% NaOH at 15 V for 5 min
FO-W	8 min

Treatment time and temperature for the oxidation of metal anodes are shown. The applied voltage and composition of electrolysis solution for electrochemical oxidation are indicated

### MFC operation

In the screening test, a plate-shaped (5 cm × 5 cm × 0.1–0.2 mm) metal anode, with or without oxidation treatment, was placed on one side of a cubic air–cathode single-chambered MFC reactor (5 cm × 5 cm × 5 cm), fabricated using a 0.8-cm-thick polycarbonate resin. The air cathode (5 cm × 5 cm), which was placed opposite to the anode, was composed of a carbon paper with 0.5 mg/cm^2^ of Pt catalyst. The reactor was filled with an artificial wastewater containing (per liter of distilled water): 1.6 g sodium acetate, 1 g meat extract, 0.3 g urea, 0.6 g NaH_2_PO_4_·2H_2_O, 0.12 g NaCl, 0.05 g KCl, 0.03 g CaCl_2_·2H_2_O, and 0.05 g MgSO_4_·7H_2_O. The MFCs were inoculated with activated sludge, collected at a livestock-wastewater treatment plant of Institute of Livestock and Grassland Science, Tsukuba, Japan, and were operated at 30 °C in a fed-batch mode. The MFCs were connected to a 13-kΩ external resistor, and the resistance value was decreased stepwise to 1.8 and 0.36 kΩ during operation.

In experiments with the selected anodes, mesh-shaped Mo and FO-W anodes (4 cm × 80 cm; with Mesh No. 100–325) were folded, and were placed in air–cathode single-chambered MFCs (5 cm × 4 cm × 3 cm). The air–cathode (4 cm × 3 cm), placed on one side of the MFC, was composed of a carbon cloth with 1 mg/cm^2^ of Pt catalyst. The MFCs were operated for 350 days in the same manner as the MFCs used in the screening test.

### Electrode surface characterization

Surface morphology of the metal anodes was characterized by scanning electron microscopy (SEM) using a JSM-5600LV (JEOL, Tokyo, Japan) instrument operated at 15 kV, followed by energy dispersive spectroscopy (EDS) to analyze their atomic composition. Molecules on the anode surface were analyzed by X-ray diffraction (XRD). Metal anodes were directly placed on a glass holder and were analyzed using RAD-X (Rigaku Co., Tokyo, Japan) under the following conditions: CuKα, 40 kV; 25 mA; divergence slit, 1°; anti-scatter slit, 1°; receiving slit, 0.15 mm; monochromator slit, 0.6 mm; scan rate, 2°/min; and scan step, 0.02°.

### Electrochemical analysis

The polarization curve for the MFCs was measured by recording the current response to a 50-mV step potential decrease, using a potentiostat/galvanostat (AutoLab PGSTAT12; Metrohm Autolab, Utrecht, The Netherlands) [16]. Each potential value was set for 50 s, and the data at the last time points were collected at each potential to allow for current stabilization. The electrical power (*P* = *IV*) was calculated using the measured current (*I*) and set potential (*V*); the power density was normalized with respect to the projected-cathode area (m^2^). The internal resistance of the MFCs was calculated based on the slope of the polarization curve [20]. To evaluate the current productivity, the polarization curves for each electrode in the MFCs were recorded by changing the electrode potential in 20-mV steps. A Pt-coated counter electrode and an Ag/AgCl reference electrode were used in this setup and each potential value was set for 20 s. Cyclic voltammetry (CV) of the anodes was performed at a scan rate of 3 mV/s in a potential window from − 0.7 to 0.2 V (vs. Ag/AgCl), using the potentiostat.

### Bacterial community structure analysis

High-throughput sequencing for the V3–V4 region of the *16S rRNA* gene was performed using the MiSeq Illumina sequencing platform (Illumina Inc., CA, USA) [21]. The anodes used in the screening test were extensively washed with distilled water, and the genomic DNA of the anode biofilms was extracted using an UltraClean™ soil DNA Isolation kit (Mo Bio Laboratories, Carlsbad, CA, USA). Sequence libraries were constructed from the genomic DNA by polymerase chain reaction, as specified by the manufacturer, and were sequenced during a 300PE MiSeq run. The read sequences were clustered into operational taxonomic units (OTUs) by the Uclust method [22] using the QIIME software [23]. Taxonomic classification, rarefaction curves, and alpha diversity were computed with QIIME. The taxonomic assignment of the major OTUs was checked using BLAST and Classifier [24]. Beta diversity analysis was performed using a weighted UniFrac distance matrix [25], and the results were visualized by principal coordinate (PCo) plot analysis. The phylogenetic tree, combined with the heat map, was calculated by the unweighted pair-group method using arithmetic averages (UPGMA) using MEGA4 [26].

## Results and discussion

### Extensive screening of metal and oxidized-metal anodes

Fourteen metals (Al, Ti, Fe, Ni, Cu, Zn, Zr, Nb, Mo, Ag, In, Sn, Ta, and W) were selected as candidate anode materials, based on the electrical conductivity of their oxidized forms and their price; of these, nine metals (Al, Ti, Fe, Ni, Cu, Zn, Mo, Sn, and W) are relatively inexpensive. To prepare the surface-oxidized-metal anodes, electrochemical oxidation and flame oxidation were performed. Since Al, Zn, Ag, In, and Sn melt upon flame oxidation, they were heat-treated instead. After the oxidation treatments, the color of the metal anodes changed to gray, dull color, brown, or blue (Additional file 1: Fig. S1). The color of In (melting point of 158 °C) did not change upon HT at 130 °C for 5 days, and the HT-In anode was, therefore, not evaluated. In the current study, no pretreatment of untreated metal anodes, such as a fluorine acid treatment to remove the metal-oxide layer that naturally forms on the metal surface in the presence of air, was conducted.

In total, 45 types of untreated and oxidized-metal anodes were evaluated using plate-shaped electrodes. Two MFC reactors were operated for each anode, and the third MFC was operated for anodes that showed a high maximum power density (> 245 mW/m^2^) in the MFCs. The anode performance was judged based on averaged readings. The raw data for power and current production are shown in Additional file 2: Fig. S2 and Additional file 3: Fig. S3, respectively, and are summarized in Table 2. The untreated Mo, EO_acid_-Mo, and EO_alk_-Mo anodes showed high average values of maximum power densities (307–344 mW/m^2^) in the screening test; there was no statistically significant difference among these Mo-based anodes. The power outputs of the FO-W, FO-Fe, FO-Mo, and Sn-based (Sn, EO_acid_-Sn, EO_alk_-Sn, and HT-Sn) anodes were relatively lower (223–278 mW/m^2^). In terms of current production, the untreated Mo and EO_acid_-Mo anodes showed high average values (1.46–1.66 A/m^2^). EO_alk_-Mo, FO-Fe, FO-W, FO-Mo, and Sn-based anodes generated relatively lower currents (0.81–1.14 A/m^2^). The remaining anodes were characterized by lower power and current generation. Flame oxidation or HT promoted the power generation of Ti, Fe, Nb, Ag, and W anodes, by 1.5–85 times, whereas electrochemical oxidation increased electricity generation of only the Ag anode (Table 2). This indicated that flame oxidation and HT are more useful than electrochemical oxidation for improving the performance of metal anodes in MFCs.

**Table 2:** Results of the screening of metal and oxidized-metal anodes

Base metal	Anode	Maximum power density (mW/m^2^)				Current productivity (A/m^2^)^a^			
		Reactor 1	Reactor 2	Reactor 3	Average ± SD	Reactor 1	Reactor 2	Reactor 3	Average ± SD
Al	No treatment	140	269	190	200 ± 65	0.53	0.80	0.58	0.64 ± 0.14
	EO_acid_-Al	141	207		174	0.43	0.46		0.45
	HT-Al	0.01	0.02		0	0.00	0.00		0.00
Ti	No treatment	28	4		16	0.02	0.05		0.04
	EO_acid-_Ti	2	5		4	0.01	0.02		0.02
	FO-Ti	162	90		126	0.59	0.38		0.49
Fe	No treatment	178	186		182	0.39	0.70		0.55
	EO_alk_-Fe	134	219		177	0.38	0.81		0.60
	FO-Fe	289	261	278	276 ± 14	1.01	0.91	1.05	0.99 ± 0.07
Ni	No treatment	165	130		148	0.43	0.41		0.42
	EO_acid_-Ni	142	11		77	0.11	0.01		0.06
	EO_alk_-Ni	120	131		126	0.08	0.27		0.18
	FO-Ni	162	110		136	0.42	0.21		0.32
Cu	No treatment	5	9		7	0.00	0.00		0.00
	EO_acid_-Cu	26	14		20	0.00	0.00		0.00
	FO-Cu	39	23		31	0.09	0.00		0.05
Zn	No treatment	192	156		174	0.16	0.28		0.22
	EO_alk_-Zn	120	152		136	0.18	0.18		0.18
	HT-Zn	160	88		124	0.36	0.08		0.22
Zr	No treatment	73	55		64	0.21	0.18		0.20
	EO_alk_-Zr	16	9		13	0.04	0.02		0.03
	FO-Zr	7	3		5	0.03	0.13		0.08
Nb	No treatment	1.3	0.8		1	0.01	0.01		0.01
	EO_acid_-Nb	0.4	0.5		0	0.01	0.00		0.01
	FO-Nb	68	111		90	0.17	0.27		0.22
Mo	No treatment	397	269	366	344 ± 67	2.15	1.08	1.76	1.66 ± 0.54
	EO_acid_-Mo	373	265	331	323 ± 54	1.76	1.13	1.50	1.46 ± 0.32
	EO_alk_-Mo	293	312	315	307 ± 12	0.93	1.09	1.40	1.14 ± 0.24
	FO-Mo	209	295	279	261 ± 46	0.67	1.09	0.96	0.91 ± 0.22
Ag	No treatment	71	135		103	0.20	0.14		0.17
	EO_acid_-Ag	196	90		143	0.59	0.35		0.47
	EO_alk_-Ag	198	242		220	0.60	0.85		0.73
	HT-Ag	238	218		228	0.49	0.59		0.54
In	No treatment	204	209		207	0.57	0.53		0.55
	EO_acid_-In	195	208		202	0.47	0.63		0.55
Sn	No treatment	279	183	265	242 ± 52	1.15	0.87	1.01	1.01 ± 0.14
	EO_acid_-Sn	198	247	245	230 ± 28	0.55	1.03	0.84	0.81 ± 0.24
	EO_alk_-Sn	287	171	286	248 ± 67	1.19	1.18	1.10	1.16 ± 0.05
	HT-Sn	259	238	171	223 ± 46	0.95	0.88	0.72	0.85 ± 0.12
Ta	No treatment	5	2		4	0.03	0.01		0.02
	EO_acid_-Ta	0	1		1	0.00	0.01		0.01
W	No treatment	73	72		73	0.00	0.00		0.00
	EO_acid_-W	68	73		71	0.00	0.00		0.00
	EO_alk_-W	77	71		74	0.00	0.09		0.05
	FO-W	253	293	288	278 ± 22	1.09	0.62	1.03	0.91 ± 0.26

^a^Current generation of the metal anodes at − 0.3 V (vs. Ag/AgCl) in a potentiostatic test

Heterogeneity in power generation was observed among the MFCs equipped with the same anode in the screening test. The cathodes used were handmade, and thus, the coating of the Pt catalyst on the surface might be slightly non-uniform among the cathodes. The bacterial community structure of activated sludge used as the inoculum might not always be identical in each experiment. Especially, based on our experiences, plate-shaped metal electrodes tended to display a larger variation in the current-generation data, as compared to mesh-shaped metal electrodes, probably due to inadequate bacterial adhesion to the smooth and flat surface of the plate-shaped electrode in the start-up period. Since mesh-shaped electrodes were commercially not available for some metals, plate-shaped electrodes were used in the screening test. These factors are likely to result in heterogeneity. The untreated Mo anode showed the highest average value of power output; however, statistically, the power generation was not significantly higher than those of the top anodes of the other metals, FO-W, FO-Fe, and EO_alk_-Sn anodes, due to data variation. Hence, in conclusion, the screening test revealed that Mo, W, Fe, and Sn are high-performance MFC anode materials. Mo and Sn anodes did not require oxidation treatment to generate increased power output, whereas W and Fe anodes needed flame oxidation treatment. MFC anodes containing Fe [16, 27, 28] and Sn [29, 30] have been characterized, but the untreated Mo and FO-W anodes have not been reported thus far. Therefore, the Mo and FO-W anodes were selected for further analyses.

### Surface characteristics of the Mo and FO-W anodes

**Fig. 1:**
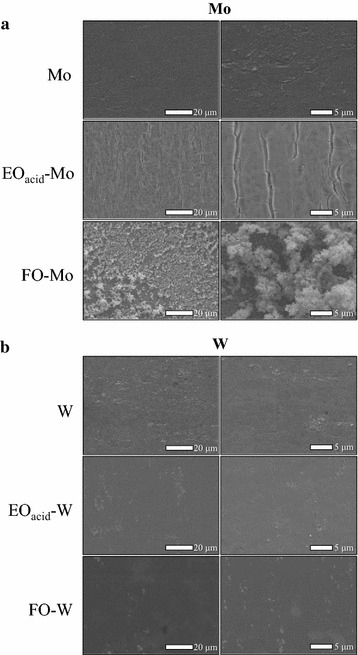
SEM images of the Mo (**a**)- and W (**b**)-based anodes

Surface morphology of the untreated Mo and FO-W anodes was analyzed using SEM (Fig. 1). For comparison, EO_acid_-Mo, FO-Mo, untreated W, and EO_acid_-W anodes were also analyzed. The surface of the untreated Mo anode was flat and smooth, but developed cleft-like alligatoring upon electrochemical oxidation. The surface of the FO-Mo anode was textured and heterogeneous. The W-based anodes (W, EO_acid_-W, and FO-W) displayed similar surface morphologies, with smooth and flat surfaces. SEM–EDS analysis revealed the presence of oxygen (4.1%) on the surface of the untreated Mo anode (Table 3), even though no oxidation treatment was performed; this indicated the presence of a Mo-oxide layer. Conversely, oxygen was not detected on the untreated W anode surface. Electrochemical oxidation of the Mo anode slightly increased the percentage of oxygen to 5.1%. Flame oxidation of the Mo anode increased it even more, to 9.4%, suggesting that the Mo-oxide layer of the FO-Mo anode was thicker than that of the untreated and EO_acid_-Mo anodes. Since the conductivity of Mo oxide (MoO_3_) is low (ca. 10^−5^ S/cm) [31], the thick oxide layer could have decreased the power output of the FO-Mo anode, which was lower than that of the untreated Mo and EO_acid_-Mo anodes in the screening test. Electrochemical and flame oxidations of the W anode increased the percentage of oxygen on the surface to the similar levels of 1.2–1.5%; this implied that the thicknesses of the W-oxide layers on EO_acid_-W and FO-W anodes were similar. Oxide film on the metal surface, generated by electrochemical oxidation, seems to contain not only oxide but also hydroxide, forming oxyhydroxide [32]. We speculate that the lower power generated with the EO_acid_-W anode (lower than with the FO-W anode) might be linked to the difference in the hydroxide content of the oxide layers.

**Table 3:** Atomic composition of the surface of the Mo- and W-based anodes, as determined by SEM–EDS

Base metal	Anode	Mo (%)	W (%)	O (%)
Mo	No treatment	95.9		4.1
	EO_acid_-Mo	94.9		5.1
	FO-Mo	90.6		9.4
W	No treatment		100	0
	EO_acid_-W		98.8	1.2
	FO-W		98.5	1.5

The values are shown as % weight of atoms

**Fig. 2:**
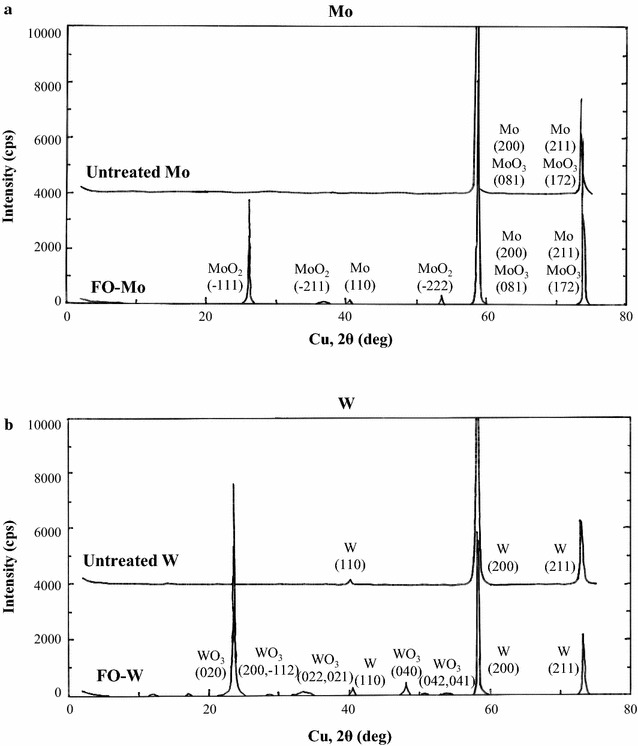
XRD profiles of the Mo (**a**) and W (**b**) anodes with or without treatment by flame oxidation

The surface of the anodes was further analyzed by XRD to determine their molecular composition (Fig. 2). Clear peaks for Mo and MoO_3_ were detected in the XRD profile of the untreated Mo anode, and no peak for MoO_2_ was observed. Peaks for Mo, MoO_2_, and MoO_3_ were observed in the profile of the FO-Mo anode. In the profile of the untreated W anode, no W-oxide peak was detected; peaks for W and WO_3_ but not WO_2_ were observed in the profile of the FO-W anode. In summary, the surface of the untreated Mo anode was naturally oxidized in the presence of air to form the MoO_3_ layer; the WO_3_ layer was produced by flame oxidation on the W anode surface.

Mo and W are situated in the same column (group 6) in the periodic table. WO_3_ is an *n*-type semiconductor, used in the photocatalysis and photoelectrolysis of water. Both MoO_3_ and WO_3_ are oxidation catalysts [33, 34], i.e., they can accept electrons in catalytic reactions. In addition, nitrogen- and low-valence-state Mo-doped MoO_3_ nanowires [31] and polyaniline/mesoporous WO_3_ composite [35] are effective MFC anode catalysts that need to be coated on a current collector before use. These observations imply that the catalytic properties of MoO_3_ and WO_3_ might be able to facilitate the electron transfer from *c*-type cytochromes in the cell walls of exoelectrogenic bacteria to the anodes, leading to an enhanced power generation in the MFCs. Further analyses are needed to verify this hypothesis.

### Power generation using the Mo and FO-W anodes

**Fig. 3:**
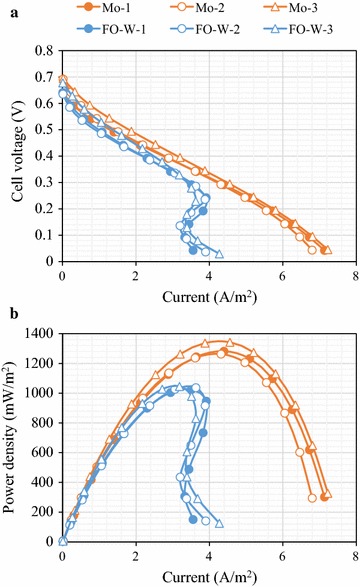
Electricity generation of MFCs equipped with the untreated Mo or FO-W anodes. Polarization curves (**a**) and power density (**b**) are shown

**Fig. 4:**
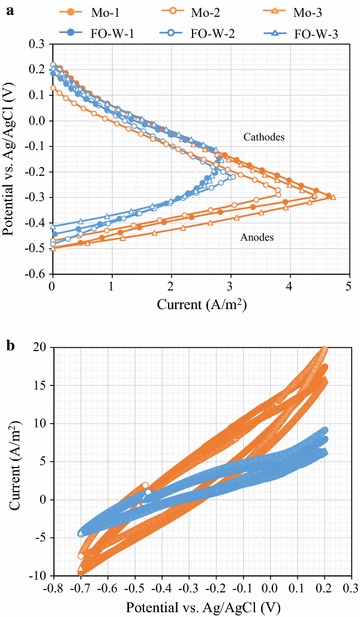
Current production of the untreated Mo and FO-W anodes. Polarization curves for the anodes and cathodes (**a**), and CV profiles (**b**) are shown

Typically, mesh-shaped anodes produce higher current in MFCs than plate-shaped anodes because of mass transfer, surface area, and biofilm formation. Therefore, the performance of the untreated Mo and FO-W anodes was further evaluated using mesh-shaped electrodes. Three MFC reactors were operated with each anode type to confirm reproducibility. The MFCs with the Mo anode produced the maximum power density of 1296 ± 42 mW/m^2^ (average ± SD), which was 25% higher than that of the FO-W anode (1036 ± 11 mW/m^2^; Fig. 3). The internal resistance of the MFCs with the Mo anode (62.3 ± 3.0 Ω) was lower than that of the FO-W anode (74.1 ± 4.6 Ω). The current response of the Mo anodes in the potentiostatic test was higher than that of FO-W anodes (Fig. 4a). At a potential of − 0.3 V (vs. Ag/AgCl), the current generated by the untreated Mo anodes was 4.25 ± 0.58 A/m^2^, which was 99% higher than that of FO-W anodes (2.13 ± 0.05 A/m^2^). A statistically significant difference (*p* < 0.05) was observed between the Mo and FO-W anodes in power density, internal resistance, and current generation. In CV analysis (Fig. 4b), the current response of the untreated Mo anode was also higher than that of the FO-W anode at all potentials tested (from − 0.7 to 0.2 V; vs. Ag/AgCl). No obvious peak was observed in either CV curve.

**Fig. 5:**
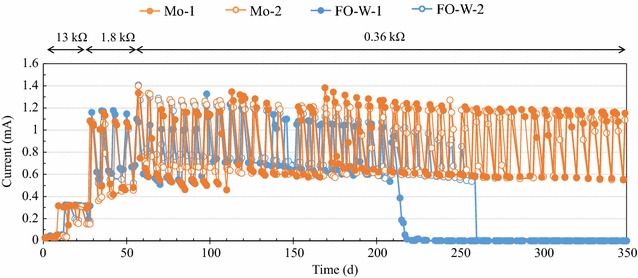
Time-courses of electricity generation in MFCs with the untreated Mo or FO-W anodes. The resistance values of external resistors connected to the MFCs are indicated

One concern with metal anodes is the corrosive nature of the operational MFC conditions. However, the Mo anodes showed excellent current production stability during long-term operation. As shown in Fig. 5, the Mo anodes continued to produce a current in the MFCs until the end of experiment (350 days), whereas electricity generation with the FO-W anodes stopped after 210–260 days because of a partial corrosion of the anodes. These results show that the Mo anode is superior to the FO-W anode with respect to both current production and long-term stability.

MFC studies have been conducted under various conditions, including various reactor configurations, medium compositions, and cathode types, all of which affect the power densities in MFCs. It is important to note that MFC outputs are usually not scalable [36]; small-sized MFC reactors tend to produce higher power densities than large-sized reactors. These factors should be considered when comparing data with the information in the literature. The reported maximum power density values using air–cathode single-chambered MFCs with a cathode area of more than 10 cm^2^, and fed defined substrates in mixed culture systems, are predominantly in the range of 400–1200 mW/m^2^ [37, 38]. The maximum power density of 1296 mW/m^2^ was achieved in the current study with the Mo anodes in MFCs with the cathode area of 12 cm^2^; this is at the top of the range, demonstrating that the Mo anode is a high-performance electrode with long-term stability.

This is the first-ever report demonstrating the usefulness of the Mo anode in MFCs. The anode is structurally quite simple and can be easily prepared by simply shaping Mo materials, such as Mo felt, Mo mesh, and Mo plate, to the desired forms. No complex processing, e.g., coating of a catalyst, is required for the preparation. The MoO_3_ layer with a putative catalytic activity forms naturally in the presence of air on the material surface. Mo is not an expensive metal, in contrast with the noble metals Pt and Au, and the commodity price of Mo is comparable with that of Cu, Ni, and Co (Additional file 4: Table S1). Many sophisticated fine-structure MFC anodes were actively developed in the last decade [7]. Most of them are carbon- or Fe-based anodes, and are prepared via complex and multistep processes, such as heating, drying, thorough mixing, and thin uniform coating of the catalyst, nafion solution, and nanostructured materials, including carbon nanotubes. Preparation of such anodes usually takes a long time (2–3 days) and requires high level of technical skill. Although they are characterized by high-performance, large-scale construction (> 1 m^3^) would be technically difficult because of the complex preparation processes. Therefore, the simplicity of the Mo anode preparation is an obvious advantage over the fine-structure anodes from a practical perspective. Another advantage of the Mo anode is its high conductivity, as compared with carbon-based anodes. The specific electrical resistibility of Mo and other metals is lower than that of graphite by three orders of magnitude (Additional file 4: Table S1). The internal resistance of MFCs increases with increasing electrode resistivity, leading to low electrical output. The high resistivity of carbonaceous anodes might lead to a non-negligible power loss in a large-scale application of MFCs, such as domestic wastewater treatment. The use of Mo anode for such large-scale applications is promising because of its conductivity and simple structure. The commodity price of Mo is higher than that of the carbon material, graphite (Additional file 4: Table S1). However, the price of manufactured Mo mesh (1400–4000 US $/m^2^) is comparable with that of carbon cloth for electrodes (1100–3300 US $/m^2^) in Japan. Moreover, Mo can be shaped to a very thin film at a thickness of 5 μm, and can coat inexpensive current collectors such as SS very thinly, by vapor deposition. These techniques could reduce the usage of Mo in anode preparation, resulting in overall cost reduction.

### Bacterial community structure of the anode biofilms

**Fig. 6:**
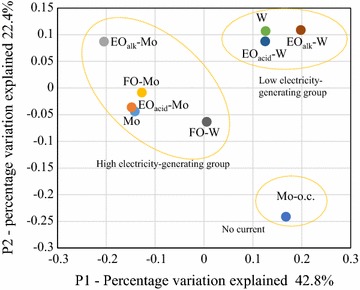
PCo plot showing the relationship between bacterial communities in biofilms formed on metal-based anodes in the MFCs

**Fig. 7:**
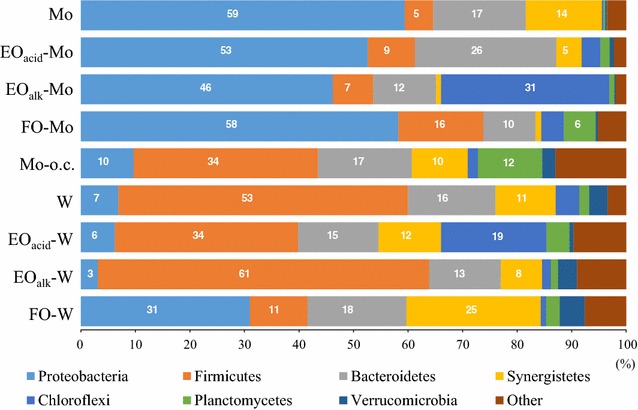
Phylum distribution within biofilm communities formed on metal-based anodes in the MFCs

Revealing the bacterial composition of an anode biofilm is essential for understanding the mechanism of electricity generation with the Mo anode. Therefore, the community structure of biofilms formed on the Mo- and W-based anodes in the screening test was analyzed using high-throughput sequencing of the retrieved *16S rRNA* genes. For comparison, a biofilm formed on an untreated Mo anode operated under open-circuit conditions (Mo–o.c.) was also analyzed. Additional file 5: Table S2 summarizes the OTU distribution and alpha diversity of these microbial communities. While Good’s coverage for all the communities was above 0.98, none of the rarefaction curves reached a plateau (Additional file 6: Fig. S4). Based on the beta diversity analysis (Fig. 6), the biofilm communities were classified into three groups; these corresponded to the electricity-generating activities observed during anode screening. The high electricity-generating group with the maximum power density (261–344 mW/m^2^) was composed of the Mo, EO_acid_-Mo, EO_alk_-Mo, FO-Mo, and FO-W communities, and was distinct from the low electricity-generating group (W, EO_acid_-W, and EO_alk_-W; 71–73 mW/m^2^) in the PCo plot. The Mo–o.c. community was distinct from both groups in the plot. Community structure analysis at the phylum level revealed that Proteobacteria was predominant (31–59%) in the high electricity-generating group; Firmicutes was abundant (34–61%) in both the low electricity-generating group and Mo–o.c. community (Fig. 7).

**Fig. 8:**
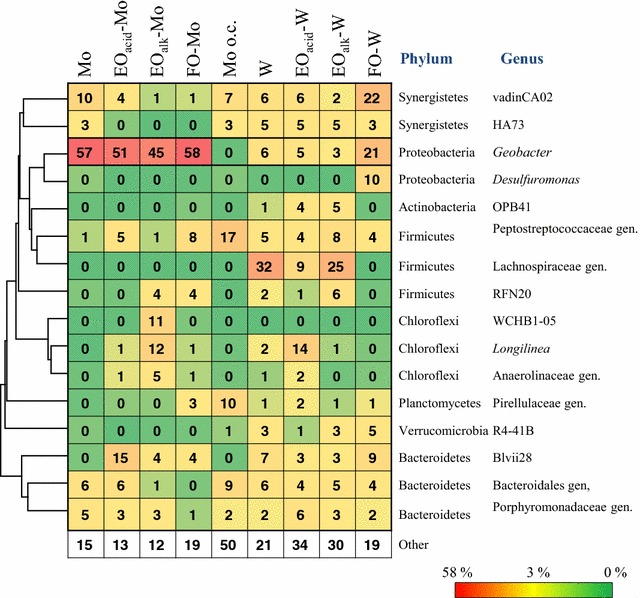
Phylogenetically clustered heat map of the major genera identified in microbial communities of the anode biofilms, based on the analysis of the *16S rRNA* gene

Genus-level analysis revealed that *Geobacter* from the phylum Proteobacteria was predominant (45–58%) in the Mo-based anodic communities with a high electricity-generating activity (Mo, EO_acid_-Mo, EO_alk_-Mo, and FO-Mo) (Fig. 8). The OTUs affiliated with *Geobacter* shared 97–99% identity with *Geobacter anodireducens* and *Geobacter hydrogenophilus* (Additional file 7: Fig. S5). The FO-W community contained *Geobacter* (21%) and another exoelectrogenic genus, *Desulfuromonas* (10%), classified in the same order Desulfuromonadales within the phylum Proteobacteria as *Geobacter*. The OTUs affiliated with *Desulfuromonas* sp. shared a 90–91% identity with *Desulfuromonas acetoxidans*, a marine anaerobe with electricity-generating activity [39]. The frequency of the exoelectrogenic genera (*Geobacter* and *Desulfuromonas*) positively correlated with the electricity-generating activity of the biofilms. The high electricity-generating group contained 31–58% of the exoelectrogenic genera, while the percentage was very low in the low electricity-generating group (3–6%) and in the Mo–o.c. community (0%). These results suggested that *Geobacter* is the main current producer in the biofilms formed on the Mo-based anodes, and that both *Geobacter* and *Desulfuromonas* are involved in the current production of the FO-W anode.

*Geobacter*-dominated biofilm community is frequently observed on carbon-based anodes. *Geobacter* was also abundant in the biofilms on the FO-SS anodes [16]. These observations suggest that *Geobacter* spp. have the ability of transferring the electrons to various types of conductive materials. Although it is not known whether *Geobacter* spp. reduce MoO_3_ and WO_3_, the bacteria could transfer the electrons to the internal regions of the Mo and FO-W anodes through the MoO_3_ and WO_3_ layers. The broad specificity and predominance of *Geobacter* in the biofilms imply the potential involvement of the bacteria in the Mo and W cycles on Earth. Mo and W are utilized as cofactors of enzymes, e.g., nitrogenases. Further studies are required to uncover the role of exoelectrogenic bacteria in the Earth metal cycles.

## Conclusions

The extensive screening test using 45 types of metal-based anodes revealed that Mo, W, Fe, and Sn are high-performance MFC anode materials. The untreated Mo anode exhibited a high maximum power density of 1273 mW/m^2^ in the MFCs. The anode is structurally quite simple and can be easily prepared by simply shaping Mo materials as required. A MoO_3_ surface layer, with a putative catalytic activity, is naturally formed in the presence of air. The anode displayed considerable stability with respect to current production during long-term operation (350 days), with no detectable corrosion. *Geobacter* was the main exoelectrogenic genus identified in the biofilm that formed on the anode. These results indicate that this anode is a promising electrode, especially suited for large-scale MFC applications, e.g., wastewater treatment.

## Additional files

**Additional file 1: Fig. S1.** Digital images of the untreated and oxidized-metal anodes.

**Additional file 2: Fig. S2.** Power density of the MFCs equipped with the untreated or oxidized-metal anodes.

**Additional file 3: Fig. S3.** Current production of the untreated and oxidized-metal anodes in the potentiostatic test.

**Additional file 4: Table S1.** Electrical conductivity and prices of anode materials.

**Additional file 5: Table S2.** Number of reads and alpha diversity analysis of microbial communities in biofilms formed on the untreated and oxidized-metal anodes in MFCs.

**Additional file 6: Fig. S4.** Rarefaction curves for the bacterial communities in biofilms formed on metal-based anodes in the MFCs.

**Additional file 7: Fig. S5.** Neighbor-joining phylogenetic trees showing the relationship between OTUs and *Geobacter* (a) or *Desulfuromonas* (b) species.

## Abbreviations

CV: cyclic voltammetry
EDS: energy dispersive spectroscopy
EO: electrochemically oxidized in acidic (EO_acid_) or alkaline (EO_alk_) electrolysis solutions
FO: flame oxidized
HT: heat treatment
MFC: microbial fuel cell
o. c.: open circuit
OTU: operational taxonomic unit
PCo: principal coordinate
SEM: scanning electron microscopy
SS: stainless steel
XRD: X-ray diffraction

## References

[1] Rabaey K, Verstraete W (2005). Microbial fuel cells: novel biotechnology for energy generation. Trends Biotechnol.

[2] Logan BE, Regan JM (2006). Electricity-producing bacterial communities in microbial fuel cells. Trends Microbiol.

[3] Lovley DR (2006). Microbial fuel cells: novel microbial physiologies and engineering approaches. Curr Opin Biotechnol.

[4] Lefebvre O, Uzabiaga A, Chang IS, Kim BH, Ng HY (2011). Microbial fuel cells for energy self-sufficient domestic wastewater treatment—a review and discussion from energetic consideration. Appl Microbiol Biotechnol.

[5] Schroder U (2007). Anodic electron transfer mechanisms in microbial fuel cells and their energy efficiency. Phys Chem Chem Phys.

[6] Zhou MH, Chi ML, Luo JM, He HH, Jin T (2011). An overview of electrode materials in microbial fuel cells. J Power Sources.

[7] Wei J, Liang P, Huang X (2011). Recent progress in electrodes for microbial fuel cells. Bioresour Technol.

[8] Baudler A, Schmidt I, Langner M, Greiner A, Schroder U (2015). Does it have to be carbon? Metal anodes in microbial fuel cells and related bioelectrochemical systems. Energy Environ Sci.

[9] Qiao Y, Li CM, Bao SJ, Bao QL (2007). Carbon nanotube/polyaniline composite as anode material for microbial fuel cells. J Power Sources.

[10] Yong YC, Dong XC, Chan-Park MB, Song H, Chen P (2012). Macroporous and monolithic anode based on polyaniline hybridized three-dimensional graphene for high-performance microbial fuel cells. ACS Nano.

[11] Zhang YZ, Mo GQ, Li XW, Zhang WD, Zhang JQ, Ye JS, Huang XD, Yu CZ (2011). A graphene modified anode to improve the performance of microbial fuel cells. J Power Sources.

[12] Qiao Y, Bao SJ, Li CM, Cui XQ, Lu ZS, Guo J (2008). Nanostructured polyaniline/titanium dioxide composite anode for microbial fuel cells. ACS Nano.

[13] Ketep SF, Bergel A, Calmet A, Erable B (2014). Stainless steel foam increases the current produced by microbial bioanodes in bioelectrochemical systems. Energy Environ Sci.

[14] Pocaznoi D, Calmet A, Etcheverry L, Erable B, Bergel A (2012). Stainless steel is a promising electrode material for anodes of microbial fuel cells. Energy Environ Sci.

[15] Guo K, Donose BC, Soeriyadi AH, Prevoteau A, Patil SA, Freguia S, Gooding JJ, Rabaey K (2014). Flame oxidation of stainless steel felt enhances anodic biofilm formation and current output in bioelectrochemical systems. Environ Sci Technol.

[16] Yamashita T, Ishida M, Asakawa S, Kanamori H, Sasaki H, Ogino A, Katayose Y, Hatta T, Yokoyama H (2016). Enhanced electrical power generation using flame-oxidized stainless steel anode in microbial fuel cells and the anodic community structure. Biotechnol Biofuels.

[17] Lovley DR, Giovannoni SJ, White DC, Champine JE, Phillips EJP, Gorby YA, Goodwin S (1993). *Geobacter metallireducens* gen. nov. sp. nov., a microorganism capable of coupling the complete oxidation of organic compounds to the reduction of iron and other metals. Arch Microbiol.

[18] Gadd GM (2010). Metals, minerals and microbes: geomicrobiology and bioremediation. Microbiology.

[19] Lovley DR (1993). Dissimilatory metal reduction. Annu Rev Microbiol.

[20] Logan BE, Hamelers B, Rozendal RA, Schrorder U, Keller J, Freguia S, Aelterman P, Verstraete W, Rabaey K (2006). Microbial fuel cells: methodology and technology. Environ Sci Technol.

[21] Kozich JJ, Westcott SL, Baxter NT, Highlander SK, Schloss PD (2013). Development of a dual-index sequencing strategy and curation pipeline for analyzing amplicon sequence data on the MiSeq Illumina sequencing platform. Appl Environ Microbiol.

[22] Edgar RC (2010). Search and clustering orders of magnitude faster than BLAST. Bioinformatics.

[23] Caporaso JG, Kuczynski J, Stombaugh J, Bittinger K, Bushman FD, Costello EK, Fierer N, Pena AG, Goodrich JK, Gordon JI (2010). QIIME allows analysis of high-throughput community sequencing data. Nat Methods.

[24] Wang Q, Garrity GM, Tiedje JM, Cole JR (2007). Naive Bayesian classifier for rapid assignment of rRNA sequences into the new bacterial taxonomy. Appl Environ Microbiol.

[25] Lozupone C, Knight R (2005). UniFrac: a new phylogenetic method for comparing microbial communities. Appl Environ Microbiol.

[26] Tamura K, Dudley J, Nei M, Kumar S (2007). MEGA4: molecular evolutionary genetics analysis (MEGA) software version 4.0. Mol Biol Evol.

[27] Ji JY, Jia YJ, Wu WG, Bai LL, Ge LQ, Gu ZZ (2011). A layer-by-layer self-assembled Fe_2_O_3_ nanorod-based composite multilayer film on ITO anode in microbial fuel cell. Colloid Surf A Physicochem Eng Asp.

[28] Lowy DA, Tender LM, Zeikus JG, Park DH, Lovley DR (2006). Harvesting energy from the marine sediment-water interface II—kinetic activity of anode materials. Biosens Bioelectron.

[29] Mehdinia A, Ziaei E, Jabbari A (2014). Facile microwave-assisted synthesized reduced graphene oxide/tin oxide nanocomposite and using as anode material of microbial fuel cell to improve power generation. Int J Hydrogen Energyy.

[30] Taskan E, Hasar H (2015). Comprehensive comparison of a new tin-coated copper mesh and a graphite plate electrode as an anode material in microbial fuel cell. Appl Biochem Biotechnol.

[31] Yu MH, Cheng XY, Zeng YX, Wang ZL, Tong YX, Lu XH, Yang SH (2016). Dual-doped molybdenum trioxide nanowires: a bifunctional anode for fiber-shaped asymmetric supercapacitors and microbial fuel cells. Angew Chem Int Ed.

[32] Delplancke JL (1983). Anodic-oxidation of iron and cathodic reduction of the anodic film—a review. Surf Technol.

[33] Tatibouet JM, Germain JE (1981). A structure-sensitive oxidation reaction—methanol on molybdenum trioxide catalysts. J Catal.

[34] Alvarez-Merino MA, Ribeiro MF, Silva JM, Carrasco-Marin F, Maldonado-Hodar FJ (2004). Activated carbon and tungsten oxide supported on activated carbon catalysts for toluene catalytic combustion. Environ Sci Technol.

[35] Wang YQ, Li B, Zeng LZ, Cui D, Xiang XD, Li WS (2013). Polyaniline/mesoporous tungsten trioxide composite as anode electrocatalyst for high-performance microbial fuel cells. Biosens Bioelectron.

[36] Osman MH, Shah AA, Walsh FC (2010). Recent progress and continuing challenges in bio-fuel cells. Part II: microbial. Biosens Bioelectron.

[37] Logan BE, Wallack MJ, Kim K-Y, He W, Feng Y, Saikaly PE (2015). Assessment of microbial fuel cell configurations and power densities. Environ Sci Technol Lett.

[38] Pant D, Van Bogaert G, Diels L, Vanbroekhoven K (2010). A review of the substrates used in microbial fuel cells (MFCs) for sustainable energy production. Bioresour Technol.

[39] Bond DR, Holmes DE, Tender LM, Lovley DR (2002). Electrode-reducing microorganisms that harvest energy from marine sediments. Science.

