# Supplementary figures and images for: Molybdenum anode: a novel electrode for enhanced power generation in microbial fuel cells, identified via extensive screening of metal electrodes
Source: Biotechnol Biofuels. 2018 Feb 13;11:39. doi: 10.1186/s13068-018-1046-7 (PMC5809899; doi:10.1186/s13068-018-1046-7)

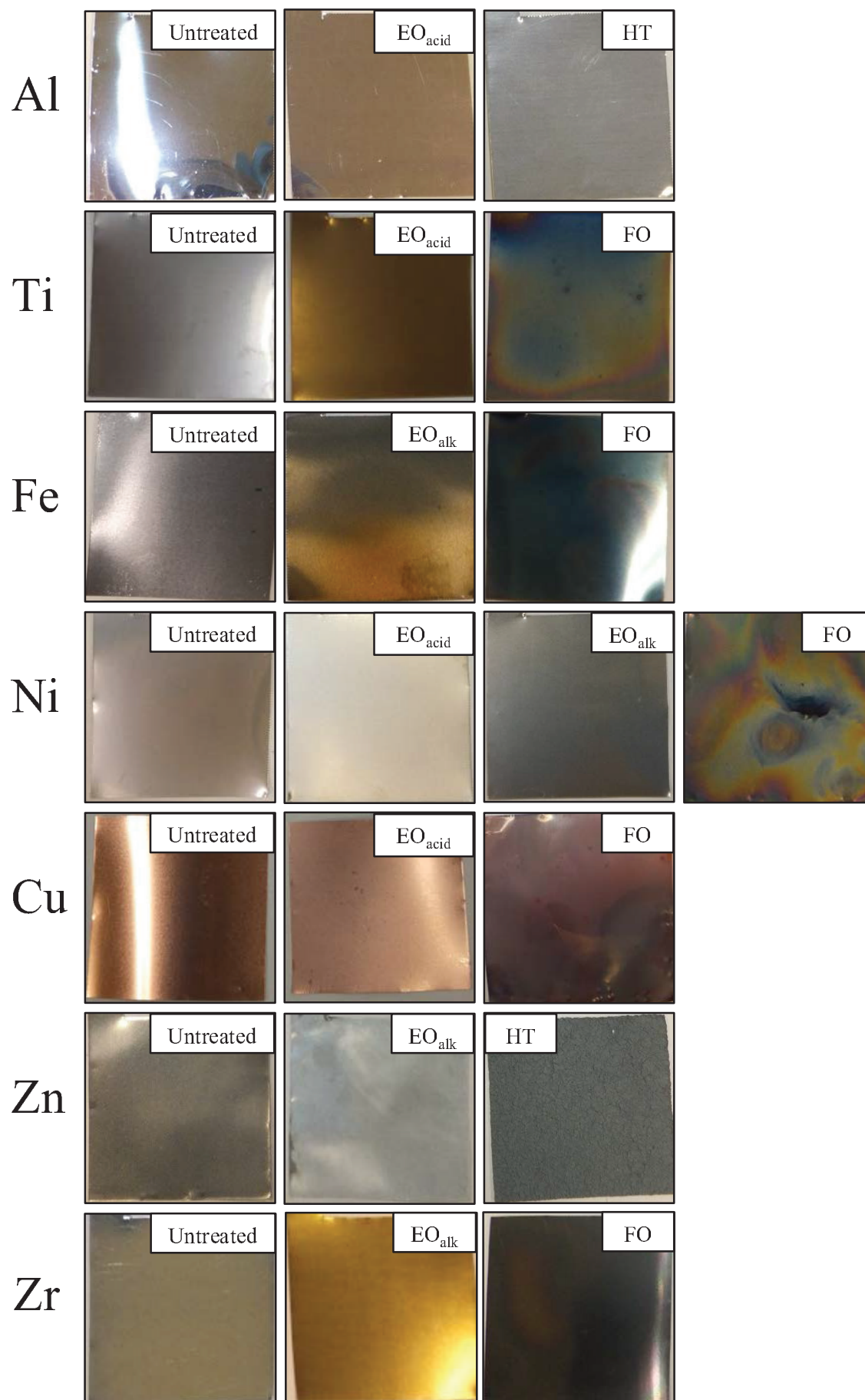

**Fig. S1. Digital images of the untreated and oxidized-metal anodes.**

Fig. S1, continued

Nb

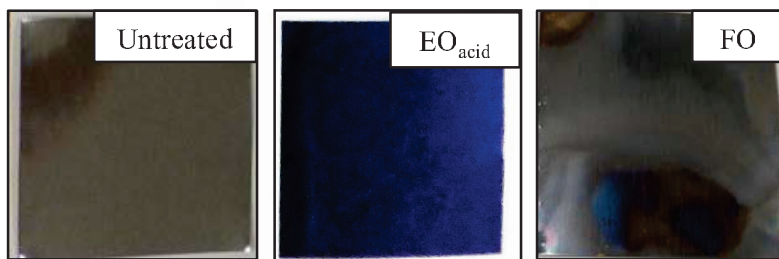

Mo

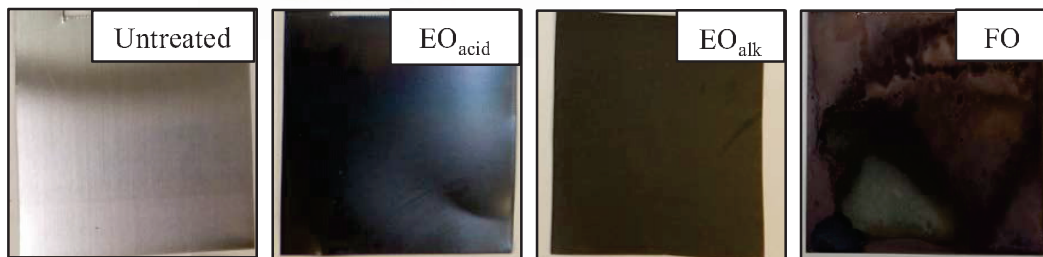

Ag

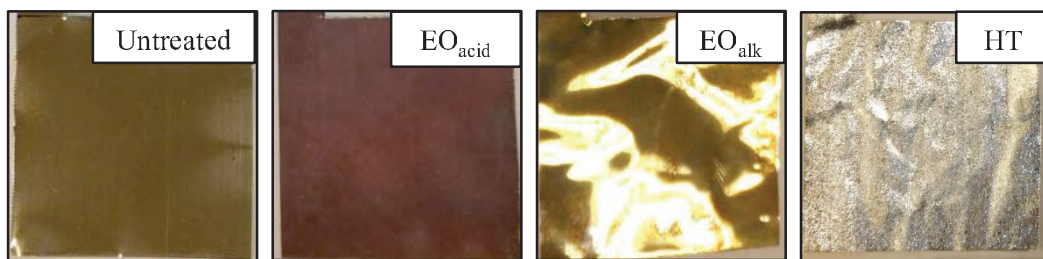

In

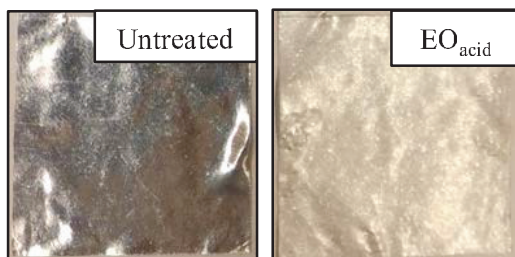

Sn

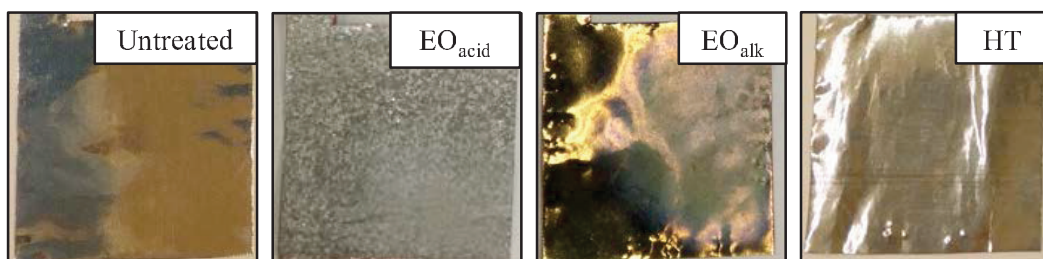

Ta

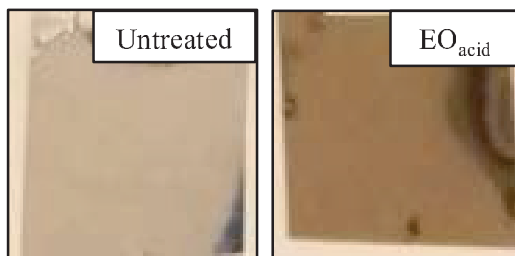

W

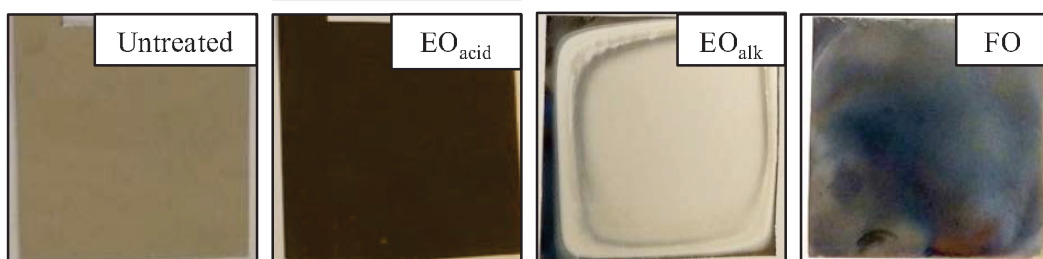

Supplement: Supplementary file 1 — Additional file 1: Fig. S1. Digital images of the untreated and oxidized-metal anodes. [file 13068_2018_1046_MOESM1_ESM.pdf]
