# Supplementary material for: Molybdenum anode: a novel electrode for enhanced power generation in microbial fuel cells, identified via extensive screening of metal electrodes
Source: Biotechnol Biofuels. 2018 Feb 13;11:39. doi: 10.1186/s13068-018-1046-7 (PMC5809899; doi:10.1186/s13068-018-1046-7)

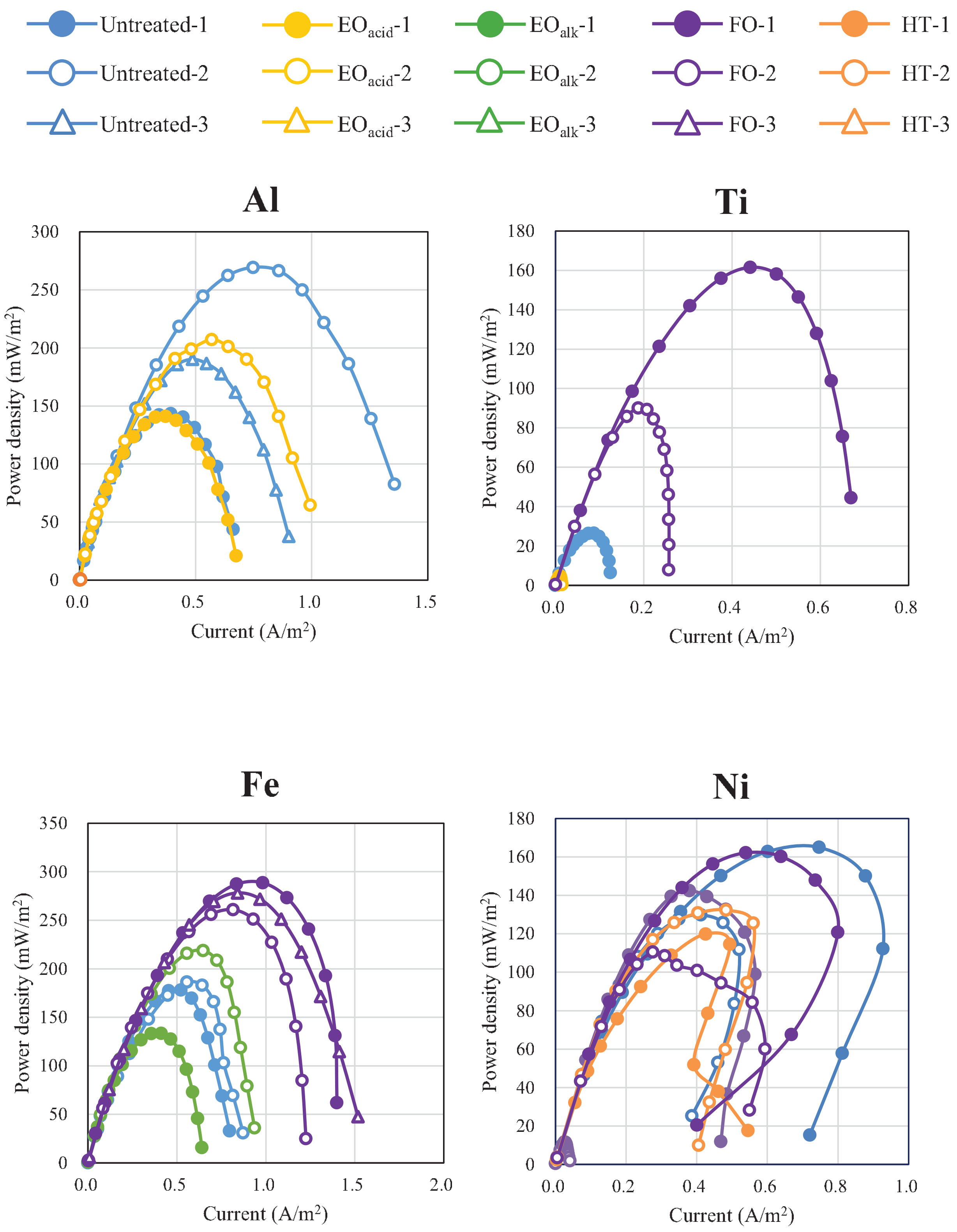

Fig. S2. Power density of the MFCs equipped with the untreated or oxidized-metal anodes.

Fig. S2, continued

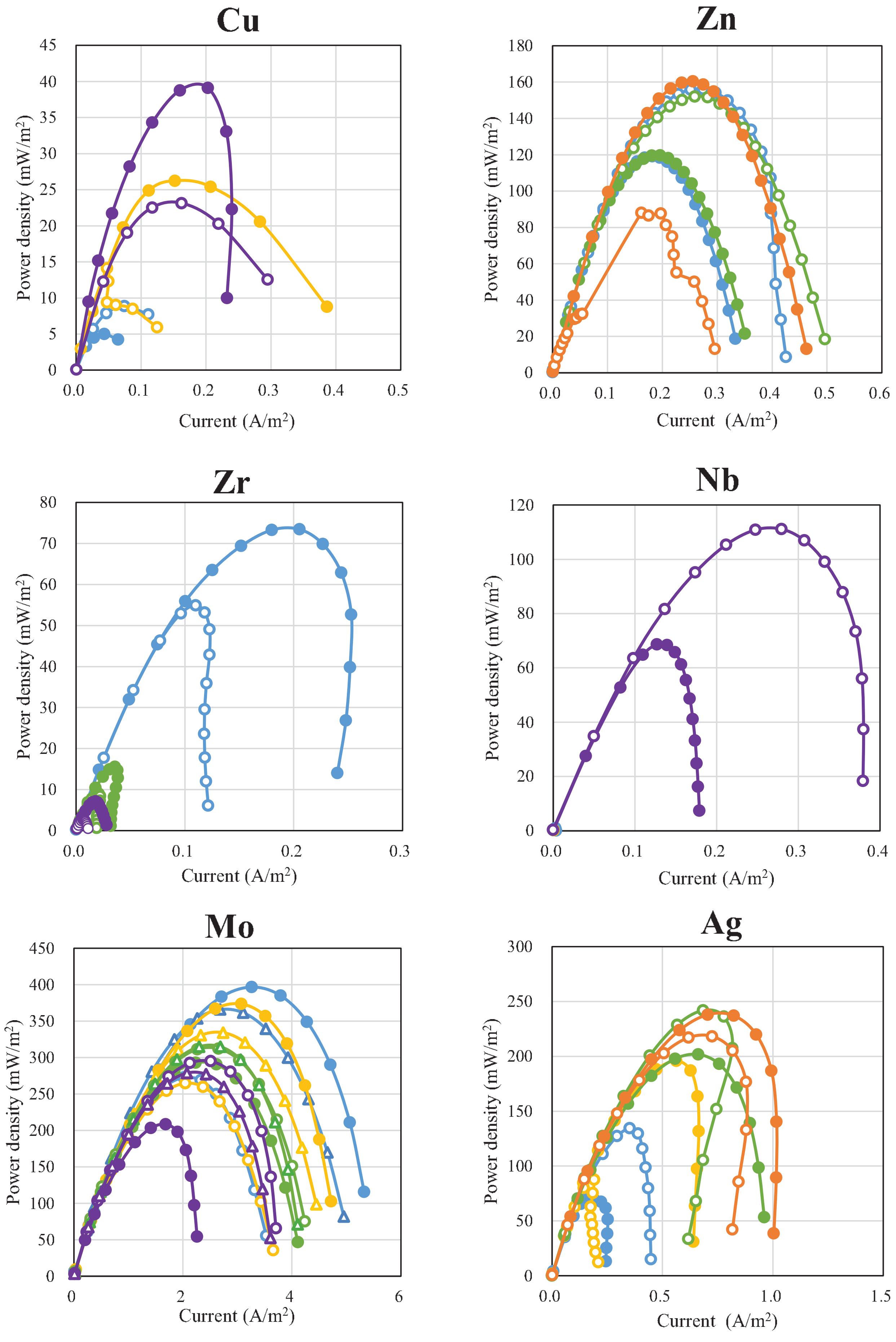

Fig. S2, continued

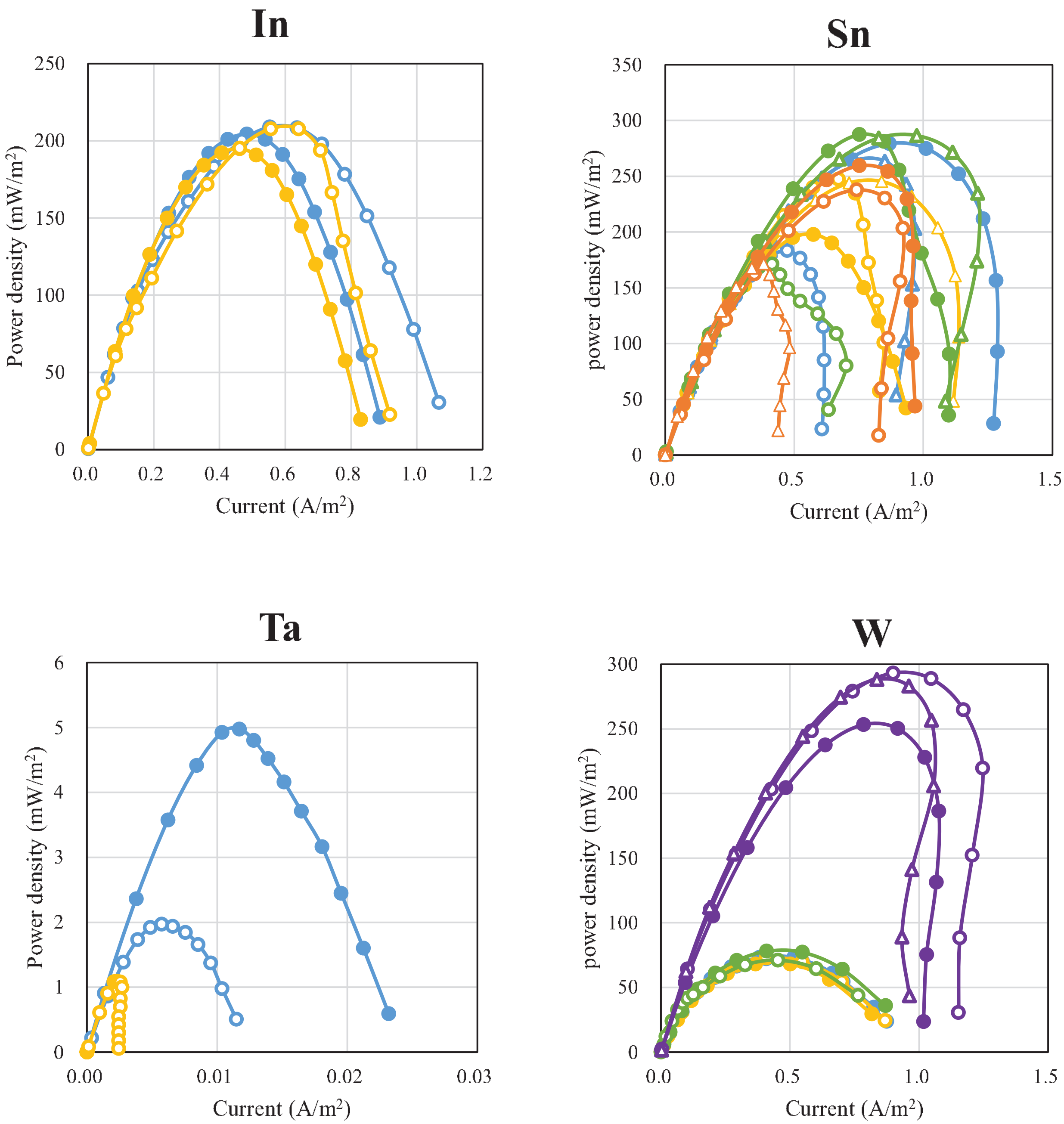

Supplement: Supplementary file 2 — Additional file 2: Fig. S2. Power density of the MFCs equipped with the untreated or oxidized-metal anodes. [file 13068_2018_1046_MOESM2_ESM.pdf]
