# Supplementary material for: Molybdenum anode: a novel electrode for enhanced power generation in microbial fuel cells, identified via extensive screening of metal electrodes
Source: Biotechnol Biofuels. 2018 Feb 13;11:39. doi: 10.1186/s13068-018-1046-7 (PMC5809899; doi:10.1186/s13068-018-1046-7)

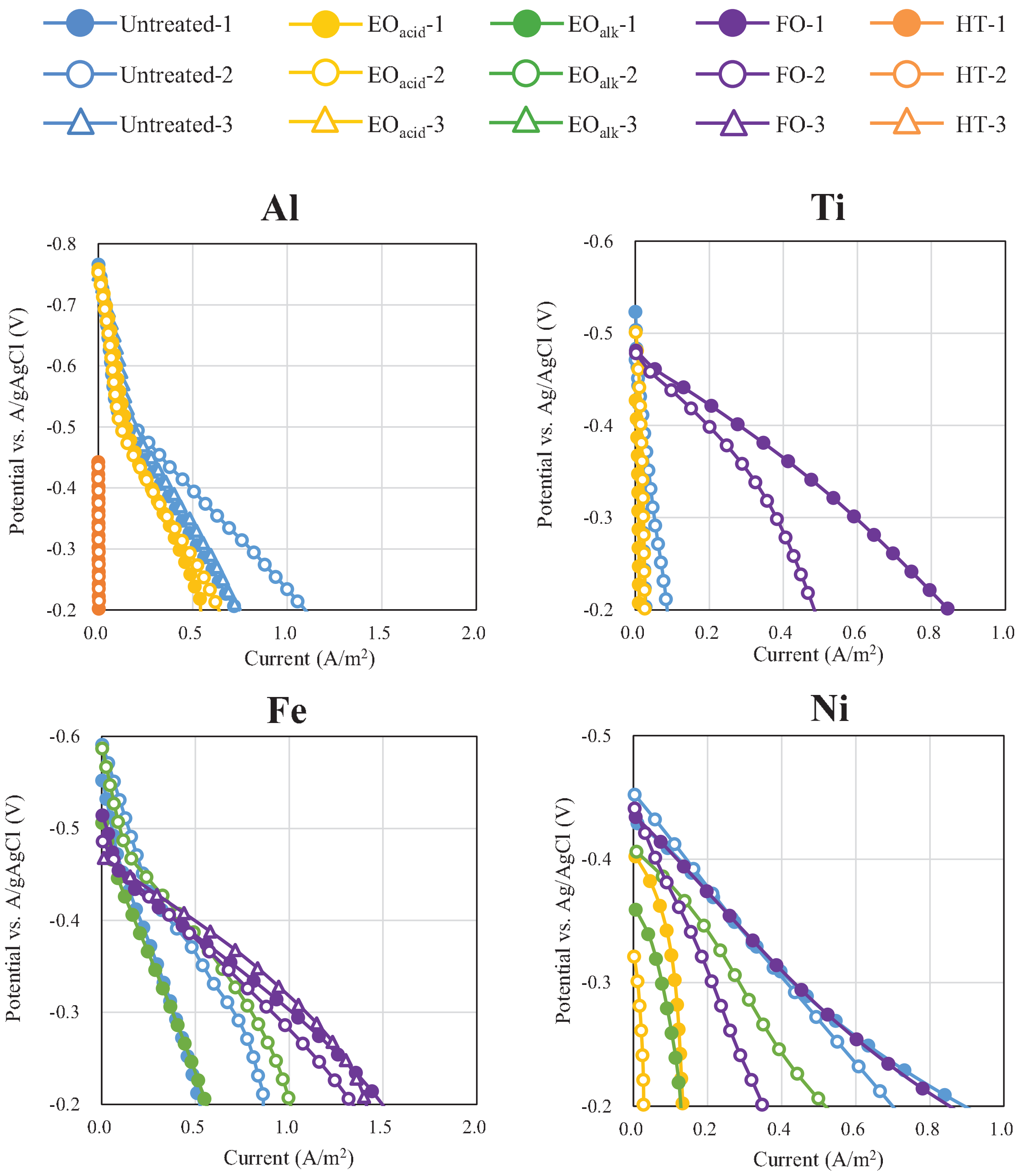

Fig. S3. Current production of the untreated and oxidized-metal anodes in the potentiostatic test.

Fig. S3, continued

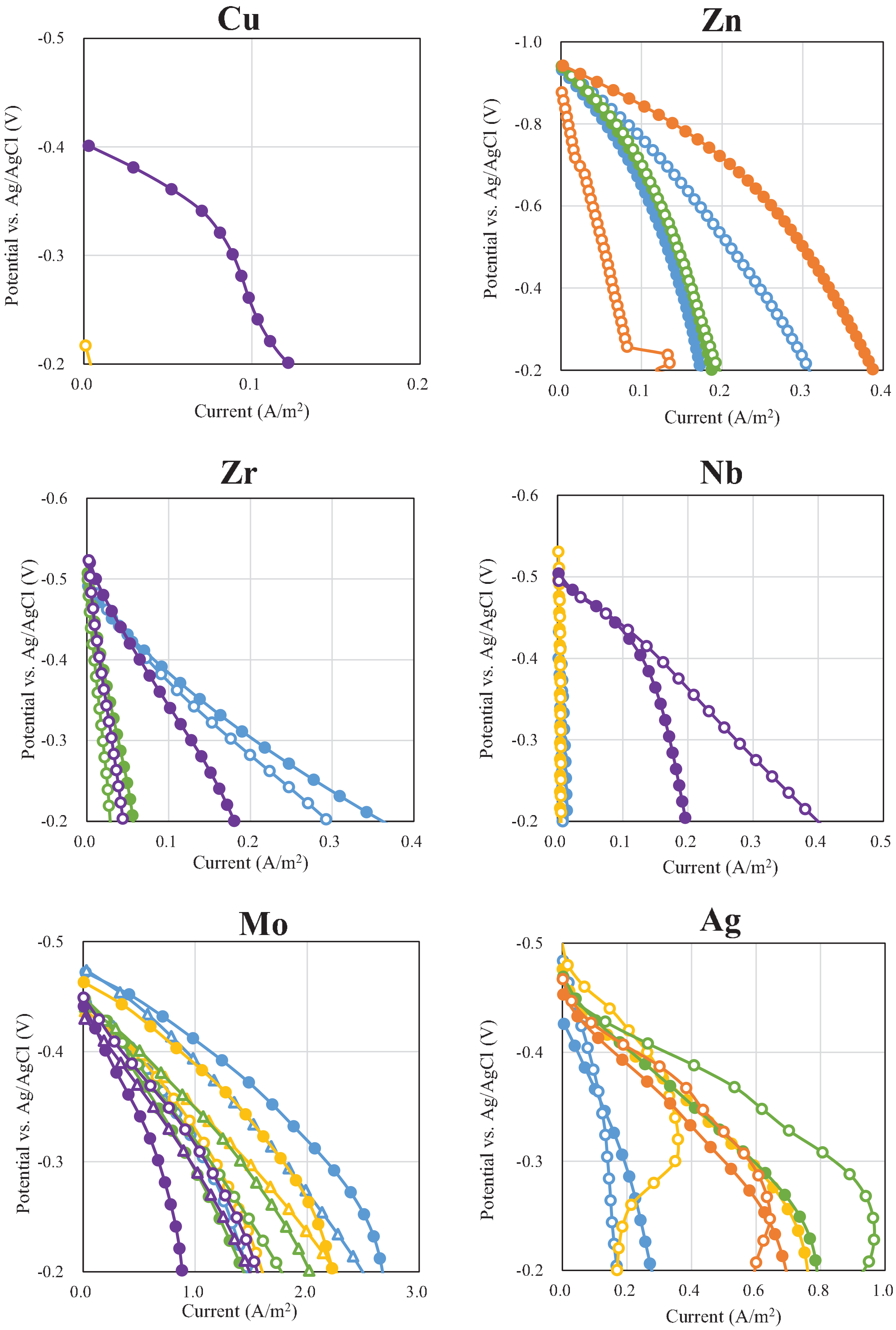

Fig. S3, continued

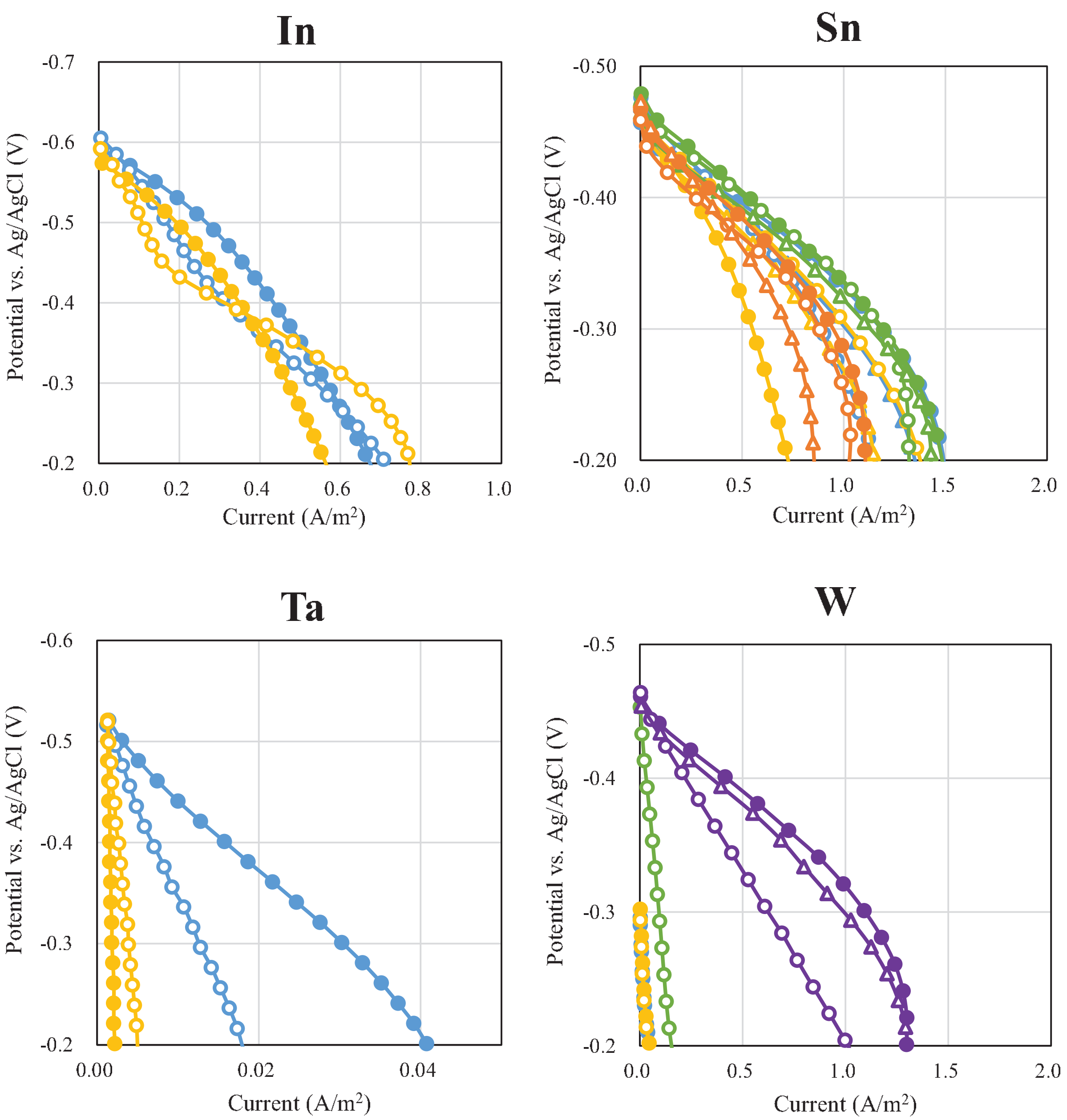

Supplement: Supplementary file 3 — Additional file 3: Fig. S3. Current production of the untreated and oxidized-metal anodes in the potentiostatic test. [file 13068_2018_1046_MOESM3_ESM.pdf]
