# Supplementary material for: Molybdenum anode: a novel electrode for enhanced power generation in microbial fuel cells, identified via extensive screening of metal electrodes
Source: Biotechnol Biofuels. 2018 Feb 13;11:39. doi: 10.1186/s13068-018-1046-7 (PMC5809899; doi:10.1186/s13068-018-1046-7)
Supplement: Supplementary file 4 — Additional file 4: Table S1. Electrical conductivity and prices of anode materials. [file 13068_2018_1046_MOESM4_ESM.pdf]

Supplementary Information

Molybdenum anode: A novel electrode for enhanced power generation in microbial fuel cells, identified via extensive screening of metal electrodes

Takahiro Yamashita[a] and Hiroshi Yokoyama[a,*]

[a]Division of Animal Environment and Waste Management Research, Institute of Livestock and Grassland Science, National Agriculture and Food Research Organization (NARO), 2 Ikenodai, Tsukuba 305-0901, Japan

[*]Correspondence

Table S1. Electrical conductivity and prices of anode materials

Anode material	Specific electric resistivity (10^{-8} Ωm)	Commodity price per ton (US $)
Cobalt	6.2	53,750[a)]
Nickel	6.8	9910[a)]
Copper	1.7	5790[a)]
Molybdenum	5.2	14,750[a)]
Silver	1.6	565,519[b)]
Tungsten	5.7	25,240[b)]
Platinum	10.6	30,825,420[b)]
Gold	2.4	39,995,886[b)]
Stainless steel (SUS304)	71	2530[c)]
Graphite	1375	1450[d)]

[a)] London metal exchange, March 2017, www.lme.com. [b)] Market website, March 2017, www.infomine.com. [c)] Global composite stainless steel price, Grade 304, MEPS international, February 2017, www.meps.co.uk. [d)] Data from (Baudler et al. 2015)
