# Supplementary material for: Molybdenum anode: a novel electrode for enhanced power generation in microbial fuel cells, identified via extensive screening of metal electrodes
Source: Biotechnol Biofuels. 2018 Feb 13;11:39. doi: 10.1186/s13068-018-1046-7 (PMC5809899; doi:10.1186/s13068-018-1046-7)
Supplement: Supplementary file 5 — Additional file 5: Table S2. Number of reads and alpha diversity analysis of microbial communities in biofilms formed on the untreated and oxidized-metal anodes in MFCs. [file 13068_2018_1046_MOESM5_ESM.pdf]

Table S2. Number of reads and alpha diversity analysis of microbial communities in biofilms formed on the untreated and oxidized-metal anodes in MFCs

Anode	Reads	OTUs	Chao1 richness	Shannon's diversity index	Abundance-based coverage estimator	Good's coverage
Mo	37,537	630	878	4.33	938	0.994
EO_{acid}-Mo	67,348	1078	1432	4.07	1457	0.995
EO_{alk}-Mo	92,629	1119	1611	3.89	1687	0.995
FO-Mo	15,383	509	824	4.26	796	0.987
Mo-o.c.	39,364	919	1177	6.50	1200	0.993
W	53,978	897	1336	5.28	1272	0.994
EO_{acid}-W	56,520	1430	2062	6.30	2125	0.990
EO_{alk}-W	110,234	1374	1812	5.58	1895	0.996
FO-W	25,553	678	1083	5.47	1101	0.989
