# Supplementary material for: Molybdenum anode: a novel electrode for enhanced power generation in microbial fuel cells, identified via extensive screening of metal electrodes
Source: Biotechnol Biofuels. 2018 Feb 13;11:39. doi: 10.1186/s13068-018-1046-7 (PMC5809899; doi:10.1186/s13068-018-1046-7)
Supplement: Supplementary file 6 — Additional file 6: Fig. S4. Rarefaction curves for the bacterial communities in biofilms formed on metal-based anodes in the MFCs. [file 13068_2018_1046_MOESM6_ESM.pdf]

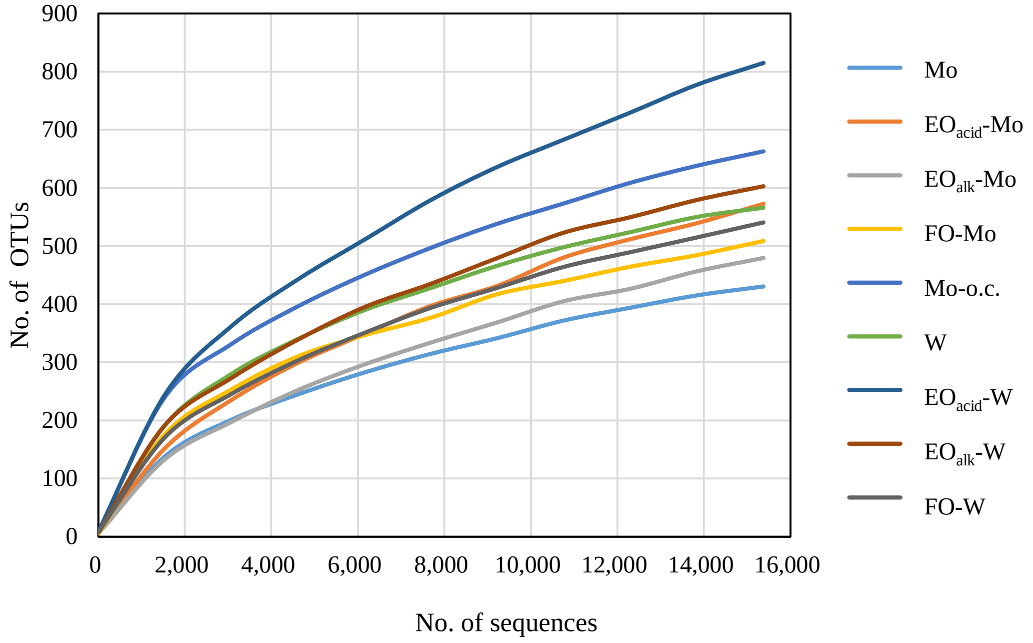

Fig. S4. Rarefaction curves for the bacterial communities in biofilms formed on metal-based anodes in the MFCs.
