# Supplementary material for: Molybdenum anode: a novel electrode for enhanced power generation in microbial fuel cells, identified via extensive screening of metal electrodes
Source: Biotechnol Biofuels. 2018 Feb 13;11:39. doi: 10.1186/s13068-018-1046-7 (PMC5809899; doi:10.1186/s13068-018-1046-7)
Supplement: Supplementary file 7 — Additional file 7: Fig. S5. Neighbor-joining phylogenetic trees showing the relationship between OTUs and Geobacter (a) or Desulfuromonas (b) species. [file 13068_2018_1046_MOESM7_ESM.pdf]

a

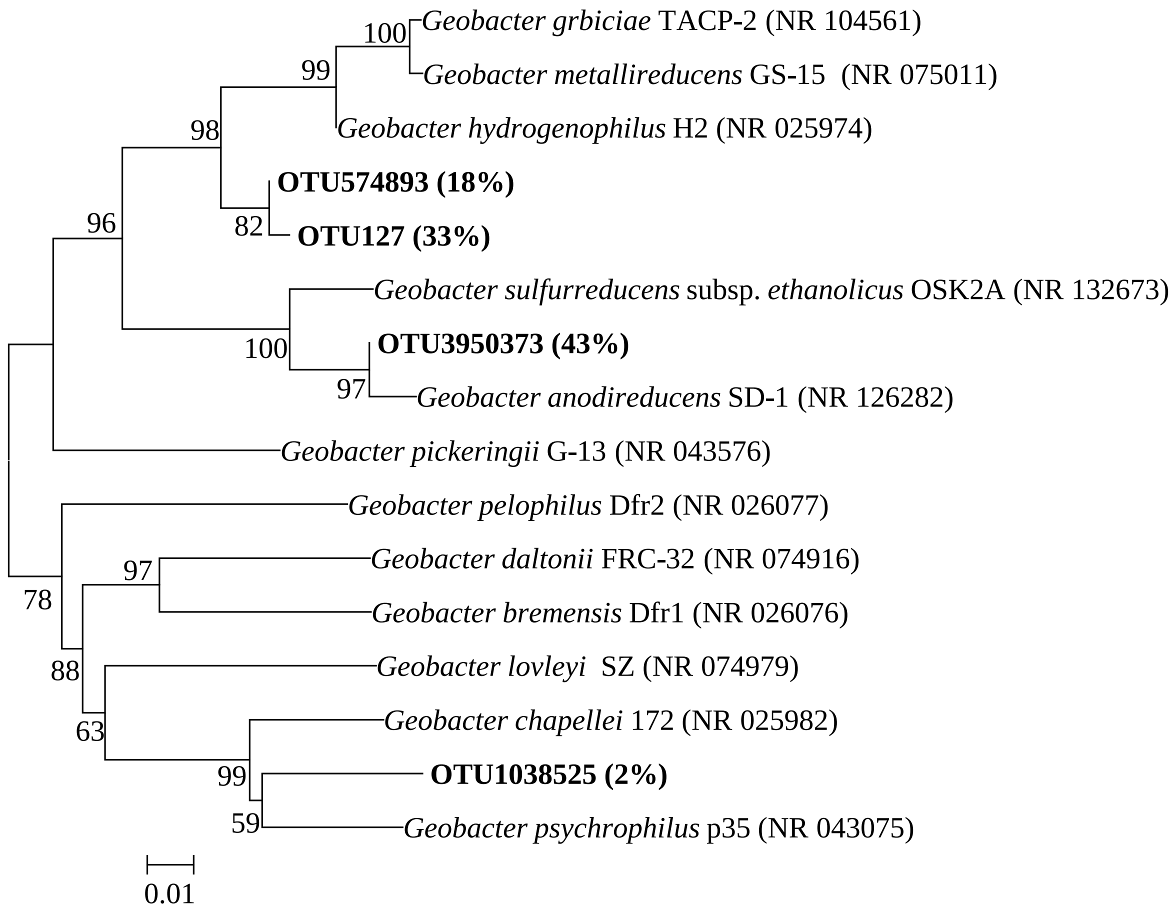

b

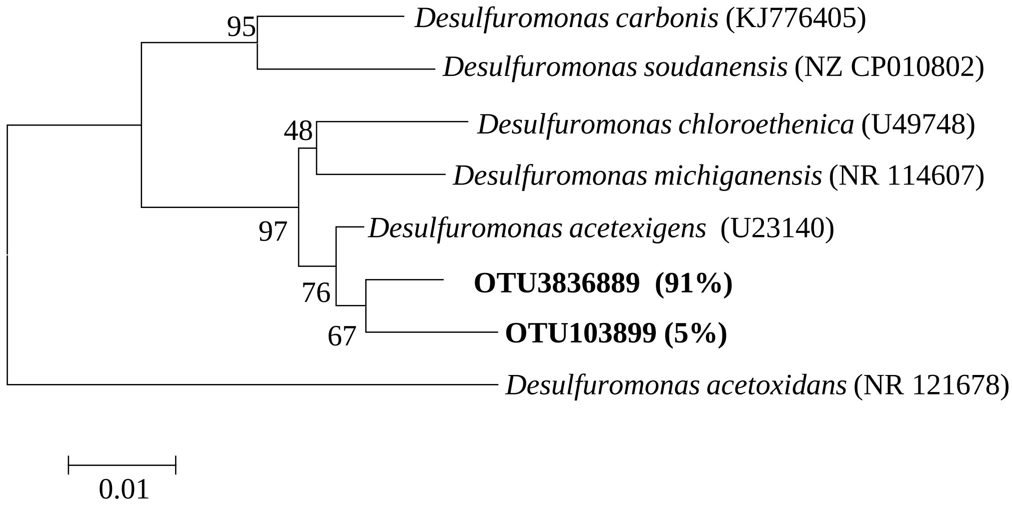

Fig. S5. Neighbor-joining phylogenetic trees showing the relationship between OTUs and *Geobacter* (a) or *Desulfuromonas* (b) species. The percentages represent the number of reads assigned to the OTUs per number of reads assigned to the genus in the anodic communities. Numbers on major branch points indicate the percent bootstrap values. The scale bars represent a 1% difference in the DNA sequences.
